# Downregulated Mucosal Autophagy, Alpha Kinase-1 and IL-17 Signaling Pathways in Active and Quiescent Ulcerative Colitis

**DOI:** 10.2147/CEG.S368040

**Published:** 2022-07-27

**Authors:** Luiza Moraes Holst, Jonas Halfvarson, Marie Carlson, Charlotte Hedin, Robert Kruse, Carl Mårten Lindqvist, Daniel Bergemalm, Sven Almér, Francesca Bresso, Maria Ling Lundström, Dirk Repsilber, Mauro D’Amato, Åsa Keita, Henrik Hjortswang, Johan Söderholm, Johanna Sundin, Hans Törnblom, Magnus Simrén, Hans Strid, Maria K Magnusson, Lena Öhman

**Affiliations:** 1 Department of Microbiology and Immunology, Institute of Biomedicine, Sahlgrenska Academy, University of Gothenburg, Gothenburg, Sweden; 2 Department of Gastroenterology, Faculty of Medicine and Health, Örebro University, Örebro, Sweden; 3 Department of Medical Sciences, Uppsala University, Uppsala, Sweden; 4 Department of Medicine Solna, Karolinska Institute, Stockholm, Sweden; 5 Department of Clinical Research Laboratory, Faculty of Medicine and Health, Örebro University, Örebro, Sweden; 6 School of Medical Sciences, Faculty of Medicine and Health, Örebro University, Örebro, Sweden; 7 Karolinska University Hospital, Gastroenterology Unit, Department of Gastroenterology, Dermatovenereology and Rheumatology, Stockholm, Sweden; 8 Clinical Epidemiology Division, Department of Medicine Solna, Karolinska Institutet, Stockholm, Sweden; 9 IKERBASQUE, Basque Foundation for Science, Bilbao, Spain; 10 Gastrointestinal Genetics Lab, CIC bioGUNE - BRTA, Derio, Spain; 11 Department of Biomedical and Clinical Sciences, Linköping University, Linköping, Sweden; 12 Department of Clinical and Experimental Science, Linköping University, Linköping, Sweden; 13 Department of Internal Medicine & Clinical Nutrition, Institute of Medicine, Sahlgrenska Academy, Gothenburg, Sweden; 14 Department of Molecular and Clinical Medicine, Institute of Medicine, Sahlgrenska Academy, University of Gothenburg, Gothenburg, Sweden; 15 Center for Functional Gastrointestinal and Motility Disorders, University of North Carolina at Chapel Hill, Chapel Hill, NC, USA; 16 Department of Internal Medicine, Södra Älvsborg Hospital, Borås, Sweden

**Keywords:** inflammatory bowel diseases, gene expression, mucosal transcriptome, homeostasis, host response

## Abstract

**Background:**

Improved mucosal immune profiling in active and quiescent colonic inflammatory bowel disease (IBD) is needed to develop therapeutic options for treating and preventing flares. This study therefore aimed to provide a comprehensive mucosal characterization with emphasis on immunological host response of patients with active ulcerative colitis (UC active), UC during remission (UC remission) and active colonic Crohn’s disease (CD active).

**Methods:**

Colonic biopsies from 47 study subjects were collected for gene expression and pathway analyses using the NanoString host-response panel, including 776 genes and 56 immune-related pathways.

**Results:**

The majority of mucosal gene expression and signaling pathway scores were increased in active IBD (n=27) compared to healthy subjects (n=10). However, both active IBD and UC remission (n=10) demonstrated decreased gene expression and signaling pathway scores related to autophagy, alpha kinase-1 and IL-17 signaling pathways compared to healthy subjects. Further, UC remission was characterized by decreased scores of several signaling pathways linked to homeostasis along with increased mononuclear cell migration pathway score as compared to healthy subjects. No major differences in the colonic mucosal gene expression between CD active (n=7) and UC (n=20) active were observed.

**Conclusion:**

This study indicates that autophagy, alpha kinase-1 and IL-17 signaling pathways are persistently downregulated in UC irrespective of disease activity. Further, UC patients in remission present a unique mucosal environment, potentially preventing patients from reaching and sustaining true homeostasis. These findings may enable better comprehension of the remitting and relapsing pattern of colonic IBD and guide future treatment and prevention of flares.

## Introduction

The natural history of inflammatory bowel disease (IBD), comprising ulcerative colitis (UC) and Crohn´s disease (CD), is marked by periods of active disease followed by periods of remission. A major challenge in the clinical management of patients with IBD is to prevent relapse and achieve mucosal healing. Over the years, numerous studies have explored the mechanisms involved in active inflammation, but far less is known of the molecular events characterizing remission. While the upregulation of a broad selection of pro-inflammatory cytokines has been extensively described in active inflammation, it is unclear whether these are completely restored during remission.^1^,^2^ Furthermore, sustained alterations related to microbial sensing, cell proliferation and resistance to apoptosis have been described in UC patients with quiescent disease, which suggests the involvement of these pathways in triggering flares.^1–4^

Differences in the clinical presentation of UC and CD, suggest distinct immunopathology profiles of the two diseases.^5^ According to traditional dogma, the mucosal inflammation in CD is considered to be T-helper (TH) 1 mediated^6^ whereas primarily thought to be TH2 driven in UC,^7^ and both conditions involve TH17 immunity.^8–11^ However, this is likely an oversimplification of a more complex and heterogeneous immunopathogenesis. When addressing potential immunological similarities and differences between UC and CD, the location of CD inflammation is important to consider, since genetic, microbiome and serology data suggest that ileal and colonic disease represent different entities.^12^,^13^ Some reports put forward that the intestinal immune profile differs between UC and colonic CD,^14^,^15^ whereas others have not detected any major differences between the two diseases.^16^,^17^ Additionally, gene signatures predicting response to anti-TNF treatment have been described in UC and Crohn’s colitis but not Crohn’s ileitis in small cohorts.^18^,^19^ In spite of their potentially different immunopathology, conventional anti-inflammatory and immunosuppressive pharmacotherapy, as well as biologic drugs targeting tumor necrosis factor (TNF), IL-12/23 and the homing receptor α_4_β_7_ show efficacy in both patient groups,^20^ supporting essential common inflammatory mediators.

During recent years, attempts to improve the characterization of the mucosal immune profiles of active and quiescent colonic IBD beyond the TH1/2/17 concept have utilized different targeted and untargeted RNA sequencing platforms, for determining mucosal gene expression.^1^,^2^,^16^,^17^,^21^ However, data are inconsistent and often lack generalizability, reproducibility and translation into signaling patterns and pathways. Characterizing the mucosal properties of IBD patients in remission will allow identification of mechanisms of importance for sustaining deep remission and preventing relapse. Furthermore, determination of colonic mucosal gene expression profiles and signaling pathways can improve our knowledge of disease phenotype/s in IBD, unifying or differentiating colonic CD and UC. In this study, we therefore aimed to provide a comprehensive characterization of colonic mucosal transcriptional profiles and signaling pathways, with emphasis on immunological host response, in patients with active UC and active colonic CD, as well as patients with UC in remission.

## Materials and Methods

### Study Subjects and Disease Activity

IBD patients were recruited at five Swedish hospitals (Sahlgrenska University Hospital, Karolinska University Hospital, Södra Älvsborg Hospital, Uppsala University Hospital and Örebro University Hospital) and healthy subjects were recruited at Sahlgrenska University Hospital.

IBD diagnosis, ie CD or UC was based on clinical, microbiological, endoscopic, histological, and radiological evaluation according to internationally accepted criteria.^22^ Patients were eligible for inclusion only if all the following criteria were fulfilled at recruitment; ≥18 years of age, confirmed diagnosis of IBD with colonic involvement for at least 3 months and ability to provide informed consent. Exclusion criteria for this cohort were: suspicion of other inflammatory colitis than IBD, including ischemic colitis and radiation colitis, previous abdominal IBD-related surgery or concurrent participation in any other study with investigational treatment within 30 days prior to inclusion. Active disease was defined as an endoscopic Mayo score ≥1 with a total Mayo score ≥3 points for UC patients, and a Harvey-Bradshaw Index (HBI) ≥5 for patients with CD. Information about histological activity from UC patients in remission was retrieved from their medical records.

Patients with active flares were assessed for response to biologic therapy 3 months post treatment start and were classified as non-responders if meeting the following criteria: (A) failure to improve partial Mayo score or HBI by ≥3 or to reach a partial Mayo score ≤2 or a HBI ≤4; and (B) failure to achieve endoscopic remission, ie endoscopic Mayo>1. In case endoscopy was not available, failure to improve faecal calprotectin levels ≥50% or decrease below 250 μg/g was used. Patients requiring steroid treatment at 3 months were also classified as non-responders. Response to treatment (responders) was defined as the absence of (A) or (B).

Adult healthy subjects were ≥ 18 years old, had no current or prior history of gastrointestinal or other chronic disorders, nor had they taken any immunosuppressive agents, antibiotics, or any other medication during the 3 months before sample collection, and they reported no current GI symptoms.

### Sample Collection

Colonic biopsies were collected during colonoscopy. Biopsies collected from patients with active disease were retrieved from inflamed sites in the colon, before start of biologic therapy. Patients in remission and healthy subjects provided biopsies from the sigmoid colon. Tissue samples were preserved in RNAlater (Invitrogen, Thermo Fisher Scientific, Belgium) and stored at −80°C, until further RNA extraction.

### RNA Extraction and Gene Profiling

Total RNA was extracted using the NucleoSpin RNA kit (Macherey-Nagel GmbH, Belgium). The purity and quantity of RNA were assessed using a NanoDrop ND-1000 spectrophotometer (NanoDrop Technologies) with 260/280 ratios of approximately 2.

Mucosal gene expression was examined using the nCounter Human Host Response panel (NanoString Technologies, Inc) at the Karolinska Institute KIGene Core Facility according to the manufacturer’s instructions. The panel included 776 transcripts associated with 5 functional themes: Host Susceptibility, Interferon Response, Innate Immune Cell Activation, Adaptive Immune Response and Homeostasis. Briefly, isolated RNA (100 ng) was hybridized with proprietary capture and reporter probes. The RNA-probe complexes were purified, immobilized, and counted. The obtained raw data were normalized against internal controls and up to 12 housekeeping genes (selected by the built-in geNorm algorithm) using the nCounter Advanced Analysis software v2 (NanoString Technologies). Thus, depending on the set of samples being compared, the housekeeping genes can vary. The Nanostring analysis also generates pathway scores by the nCounter Advanced Analysis software, calculated as the first principal component of the pathway genes’ normalized expression. Positive scores correspond to upregulation and negative scores to downregulation in a majority of the pathway genes compared with the reference group. For the Human Host Response panel, 56 pathways are generated and linked to the 5 functional themes (Supplementary Table 1).

### Biomarker Analyses

Serum concentration of high-sensitivity C-reactive protein (CRP) was analyzed in a single batch at the end of the study. Correspondingly, faecal calprotectin was extracted and analyzed with a chemiluminescent immunoassay in a single batch with the LIAISON XL analyzer, as routine tests at the Academic Laboratory, Department of Clinical Chemistry, Uppsala University Hospital, Uppsala Sweden.

### Statistical Analyses

Data and statistical analyses were performed using R 4.0.2, IBM SPSS Statistics 25 and GraphPad Prism 9 (GraphPad Software). Principal component analyses (PCA) were performed using the pca3d package in R. Volcano plots were generated using the ggplot2 and ggrepel packages in R, the log_2_-fold change of the means was calculated and differences between group s were determined using the Student-*t*-test. To correct for false-positive results in multiple comparisons, the Benjamini–Yekutieli method was used and data show *q*-values. Forest plots were generated in Microsoft Excel 2016 based on Log2 fold change values of the genes between analyzed groups. Orthogonal Projections to Latent Structures Discriminant Analyses (OPLS-DA) were implemented to correlate selected y-variables (patient groups) to x–variables (pathway scores) in linear multivariate models using SIMCA software (version 16; MKS Data Analytics Solutions). The R2Y parameter represents the goodness of fit of the model (best possible fit: R2Y = 1). The Q2 parameter represents an estimate of the predictive ability calculated by cross-validation (best possible predictive ability: Q2 = 1). For heterogeneous biological variables, a model is considered to have a good fit with an R2Y ≥0.5 and a good predictive ability with a Q2 >0.4.^23^ When focusing on pathway analysis comparing UC remission and healthy controls, a Variable Influence on Projection (VIP) >1.0 was used to select the most important pathways. Column bars in the OPLS-DA column loading plots represent mean values, and error bars show 95% confidence intervals. The significance of the model was confirmed in SIMCA through CV-ANOVA and is expressed as *p*-values.

Pathway score differences between two groups were assessed using the Mann–Whitney-*U*-test. To correct for false-positive results in multiple comparisons, the classical 1-stage method was employed, and data were then presented as q-values in the OPLS-DA column loading plots. For comparison between three groups, Kruskal–Wallis test followed by Dunn’s multiple comparisons test were used.

The level for significance was set to <0.05. For the volcano plots, a cut-off of q <0.01 was used. A power analysis to estimate the size of patient cohorts was not included in the experimental design.

## Results

### Characteristics of Study Subjects

The study cohort consisted of 37 patients with IBD and 10 healthy subjects. The IBD patients were grouped according to diagnosis and disease activity and included 20 patients with active UC (UC active), 10 patients with UC in remission (UC remission) and 7 patients with active Crohn’s colitis (CD active). Demographic data and clinical characteristics, including measures of disease activity and biopsy sites are shown in Table 1. No demographic differences were found between CD active and UC active groups except for CRP levels, which were higher in CD active (p=0.04). UC patients in remission were older than UC active (p=0.01) and had a longer disease duration (p=0.001). The healthy subjects had a median age of 31 years with a range of 21–54 years and 5 were women. The healthy subjects were younger than UC patients in remission (p=0.02). Information regarding response to biologic therapy 3 months after treatment start was retrieved from 24 patients and is shown in Supplementary Table 2. Histologic examination of biopsies from UC patients in remission showed no features of active or chronic inflammation, except for one patient showing subtle lymphocyte infiltration in isolated areas of the lamina propria.

**Table 1:** Patient Demographics and Disease Activity at Inclusion

	UC Active N=20	CD Active N=7	UC Remission N=10	p-value UC Active vs CD Active	p-value UC Active vs UC Remission
Age (median, range)	28 (21–74)	44 (23–70)	50 (24–57)	0.16	0.01
Gender (male/female)	7/13	5/2	5/5	0.18	0.46
Disease duration, years (median, range)	5 (0–33)	0 (6–50)	20 (8–43)	0.39	0.0006
Disease extent UC (E1/E2/E3)	1/6/13	NA	0/2/8	NA	0.34
Disease location CD (L2/L3)	NA	5/2	NA	NA	NA
Mayo score (median, range)	7 (4–11)^1^	NA	0 (0–0)	NA	<0.0001
Endoscopic Mayo score (0/1/2/3)	0/0/15/5	NA	10/0/0/0	NA	<0.0001
HBI (median, range)	NA	7 (5–11)^1^	NA	NA	NA
Fecal calprotectin, g/g (median, range)	772 (50–11,100)	765 (139–2320)	Not measured	0.96	NA
CRP (median, range)	2.5 (0.3–55)	7.8 (1.5–64)	Not measured	0.04	NA
Sampling location					
Right colon	1	3	0		
Transverse colon	2	0	0		
Left colon	4	0	0		
Sigmoid colon	7	2	10		
Rectum	6	2	0		
Treatment					
5-ASA	11	2	6		
Thiopurines	12	2	0		
Corticosteroids	6	4	0		
Biologics	0	0	0		
No treatment	2	2	4		

**Note**: ^1^Information for one patient is missing.

**Abbreviations**: 5-ASA, 5-aminosalicylic acid; CD, Crohn’s disease; CRP, C-reactive protein; E1, proctitis; E2, left sided colitis; E3, extensive colitis; HBI, Harvey-Bradshaw Index; L2, colonic; L3, ileolocolonic; NA, not applicable; UC, ulcerative colitis.

### Patients with Active IBD Have a Distinct Colonic Mucosal Transcriptional Profile

**Figure 1:**
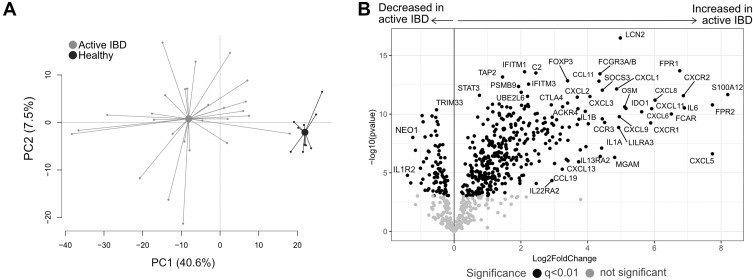
Gene expression analysis in active IBD and healthy subjects. Total RNA from mucosal biopsies was collected from inflamed sites from patients with IBD and healthy subjects. Expression of 776 genes was measured using the NanoString nCounter Host response panel. (**A**) Principal component analysis showing the IBD active group in light grey and healthy group in dark grey. (**B**) Volcano plot representing differential gene expression between IBD active vs healthy displayed as log_2_ fold change vs significance (Student’s *t*-test). False discovery rate analysis was performed using Benjamini**–**Yekutieli method, the cut-off was set to q<0.01. n=27; healthy, n=10.

**Abbreviations**: PC, principal component; IBD, inflammatory bowel disease.

To evaluate mucosal transcriptional differences between active IBD and healthy subjects, CD active and UC active patients were grouped as active IBD and examined in a PCA on all gene expressions with healthy subjects. The transcriptional profiles from inflamed and healthy colon showed a clear separation (Figure 1A) with 81 downregulated and 370 upregulated genes in IBD patients compared with healthy subjects (q<0.01) (Figure 1B).

**Figure 2:**
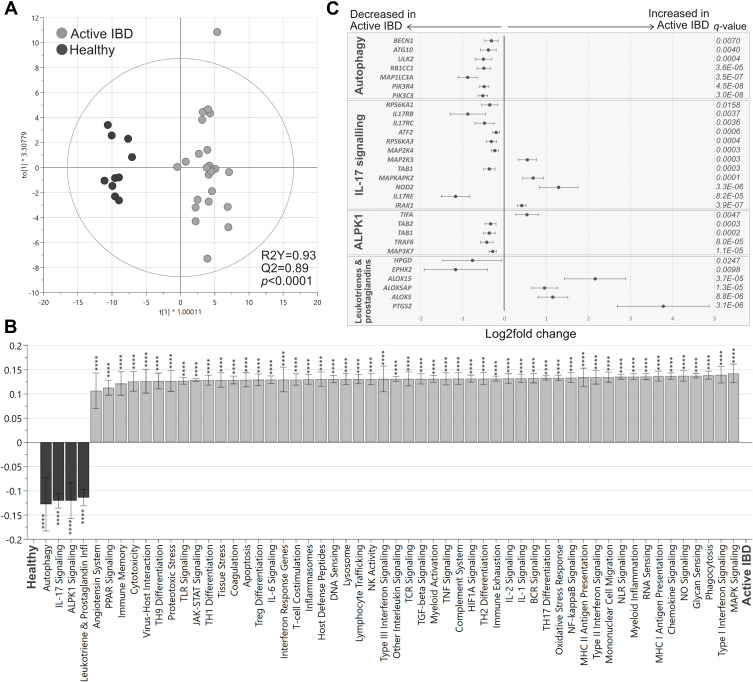
Pathway analysis in active IBD and healthy subjects. Total RNA from mucosal biopsies was collected from inflamed sites from patients with IBD and healthy subjects. Expression of 776 genes was measured using the NanoString nCounter Host response panel and pathway scores for 56 pathways were generated. (**A**) Orthogonal partial least squares discriminant analysis (OPLS-DA) score scatter plot based on pathway scores for active IBD and healthy. (**B**) OPLS-DA loading column plot depicting up- and downregulated pathways in IBD vs healthy subjects based on their pathway scores. Mann–Whitney *U*-test and false discovery rate analysis (classical 1-stage method) were used to compare the data; ****p<0.0001. (**C**) Differentially expressed genes participating in the pathways *autophagy, IL17 signaling, ALPK1* and leukotriene and prostaglandin inflammation are shown in a forest plot based on log_2_ fold change. Error bars represent 95% confidence intervals. Student’s *t*-test was used to calculate significance between the groups (p-values) and false discovery rate analysis was performed using the Benjamini**–**Yekutieli method (q-values), cut-off was set to q<0.01. n=27; healthy, n=10.

**Abbreviation**: IBD, inflammatory bowel disease.

Analyses of the pathway scores in an OPLS-DA revealed a strong separation between the groups (*p*<0.0001; Figure 2A), with most pathways scores increased in active IBD except for alpha kinase-1 (ALPK1), autophagy, IL-17 signaling and leukotriene and prostaglandin inflammation pathways, which were decreased in active IBD compared with healthy subjects (Figure 2B). Further analyses of the genes involved in the downregulated pathways showed that genes linked to prostaglandin and leukotriene synthesis (*ALOX5, ALOX15, ALOX5AP, PTGS2*), pattern recognition (*NOD2, TIFA*), TH17 differentiation (*IRAK1*) and colon carcinogenesis (*MAPKAPK2, MAP2K3*) were upregulated in active IBD (Figure 2C). In addition, genes linked to autophagy (*BEC1, ATG10, ULK2, RB1CC1, MAP1LC3A, PIK3R4, PIK3C3*), IL-17 receptor signaling cascades (*RPS6KA1, IL17RB, IL17RC, ATF2, RPS6KA3, MAP2K4*, Table 1, *IL17RE*), ALPK1 signaling (*TAB2*, Table 1, *TRAF6, MAP3K7*) and prostaglandin degradation (*HPGD, EPHX2*), were downregulated in active IBD compared with healthy subjects (Figure 2C).

### Transcriptional Profiles of Inflamed Colonic Biopsies in CD and UC are Similar and Not Affected by Pharmacotherapy

**Figure 3:**
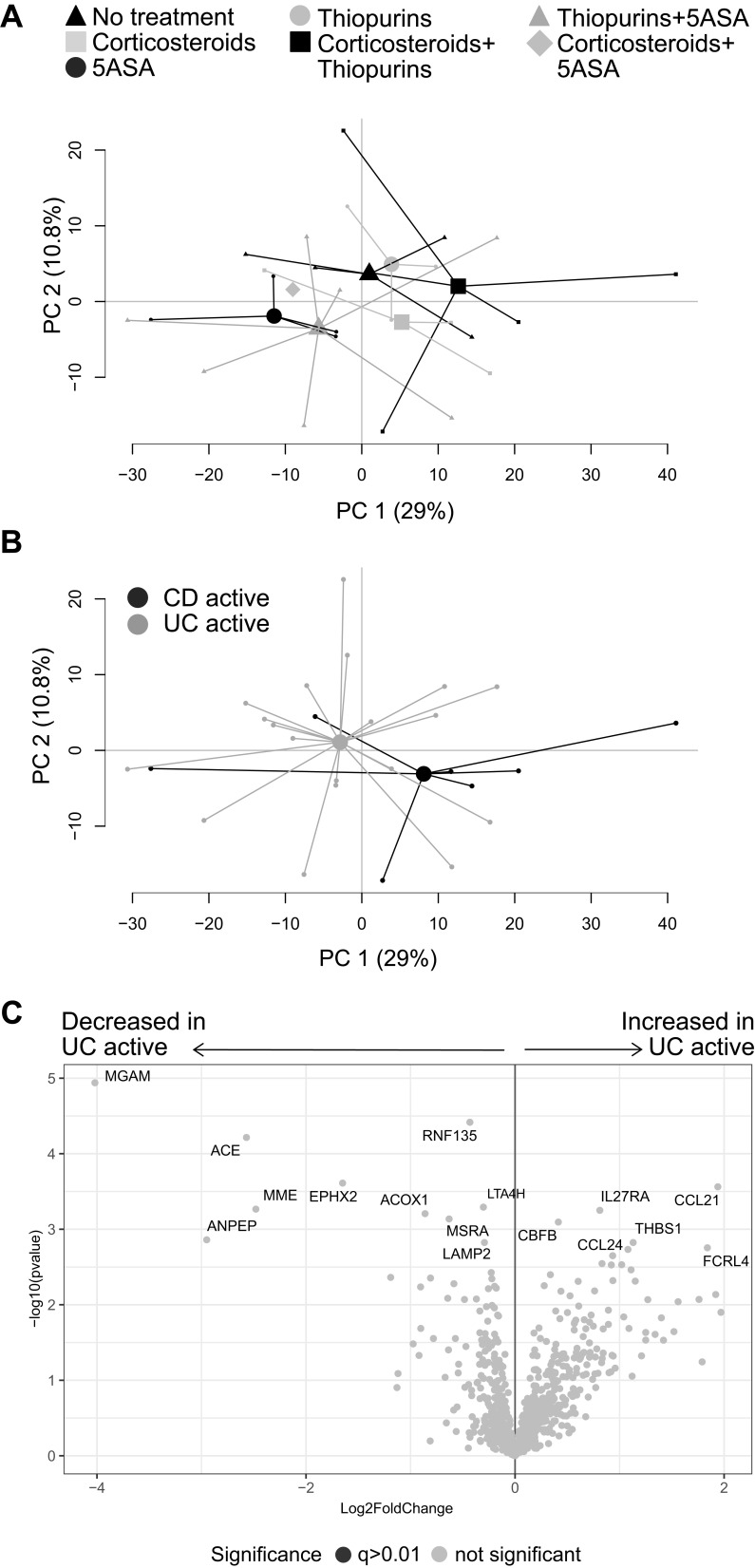
Gene expression analysis in active IBD patients. Inflamed colonic biopsies were collected from UC and CD patients with active disease and analyzed using NanoString nCounter Host Response panel for gene expression (776 genes). Principal component analysis based on the full set of genes is shown between (**A**) patients receiving no treatment, corticosteroids, 5-ASA, thiopurines, corticosteroids+thiopurines, thiopurines+5-ASA and corticosteroids+5-ASA, and (**B**) CD active vs UC active. The volcano plot (**C**) represents gene expression analysis of UC active vs CD active and show log_2_ fold change vs significance. Student’s *t*-test and false discovery rate analysis using Benjamini–Yekutieli method were used, cut-off was set to q<0.01. No treatment, n=4; Corticosteroids, n=3; 5-ASA, 5-aminosalicylic acid, n=4; Thiopurines, n=3; Corticosteroids+thiopurines, n=5; Thiopurines+5-ASA, n=7; Corticosteroids+5-ASA, n=1; CD, Crohn’s disease, n=7; UC, ulcerative colitis, n=20; PC, principal component.

To investigate if the pharmacological treatment influences mucosal gene expression, patients with active IBD were grouped according to ongoing treatments. A PCA including all genes, comparing patients receiving no treatments with those receiving 5-ASA, corticosteroids or thiopurines in mono- or combined therapy, revealed overlap between the groups (Figure 3A). Furthermore, colonic transcriptional profiles were not associated with response to biologic therapy 3 months after treatment start, as seen in a PCA based on all genes, comparing responders with non-responders (Supplementary Figure 1). Next, the transcriptional differences between active CD and active UC were analyzed in a second PCA on all gene expressions, which did not show any clear separation (Figure 3B). Differential gene expression analyses revealed 77 upregulated and 53 downregulated genes (p<0.05) when comparing UC to CD, however none survived correction for false discovery rate of q<0.01 (Figure 3C). The lowest q-value obtained was q=0.06 for maltase-glucoamylase intestinal (*MGAM*).

To evaluate if the results were affected by sampling bias, a PCA was performed on all gene expressions, grouping samples according to sampling location, but no major separation was observed (Supplementary Figure 2). Furthermore, analyses of the pathway scores in an OPLS-DA did not result in any significant components (data not shown). Collectively, these results indicate that anti-inflammatory and/or immunosuppressive treatments of patients in the present study did not affect mucosal transcriptional profiles in active IBD and that the gene expression differences between active CD and active UC in this setting were only minor.

### Colonic Mucosal Transcriptional Profiles from Active UC Patients Differ from UC Patients in Remission

**Figure 4:**
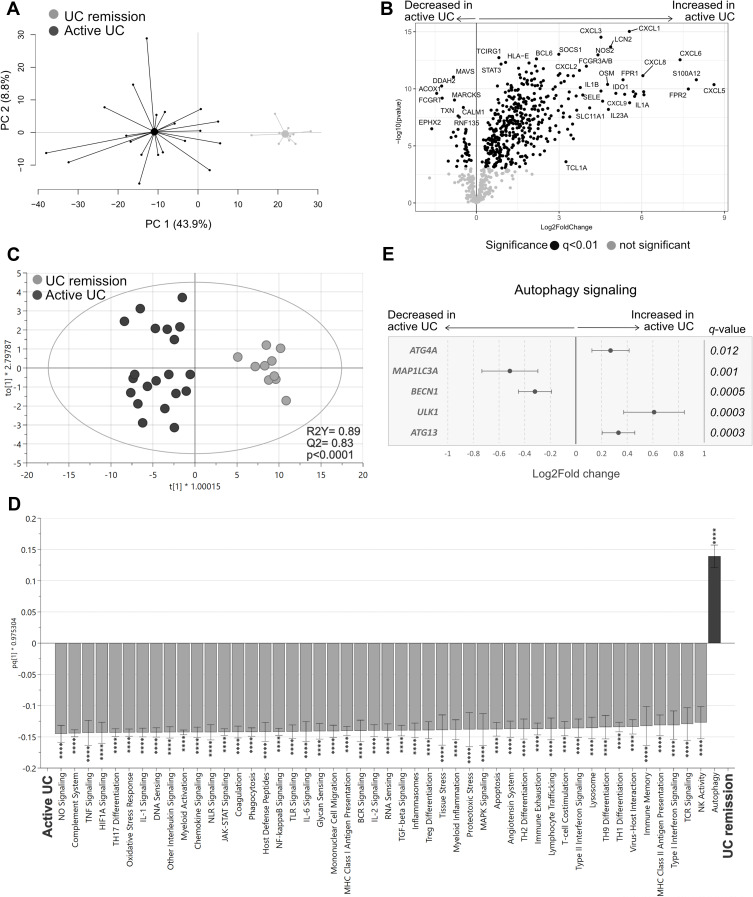
Gene expression and pathway analysis in UC patients with active disease vs UC patients during remission. Gene expression in colonic biopsies from UC patients with active disease or during remission were analyzed using NanoString nCounter Host response panel (776 genes) and pathway scores for 56 pathways were generated. (**A**) Principal component analysis based on the full set of genes genes. (**B**) Volcano plot representing differential gene expression between UC active vs UC remission displayed as log_2_ fold change vs significance. (**C**) Orthogonal partial least squares discriminant analysis (OPLS-DA) score scatter plot based on the 56 pathway scores. (**D**) OPLS-DA loading column plot depicting up- and downregulated pathways in active UC vs UC remission based on their pathway scores. (**E**) Differentially expressed genes participating in the autophagy pathway are shown in a forest plot displaying log_2_ fold change vs genes. Student’s *t*-test was used for calculating significance and false discovery rate analysis was performed using Benjamini–Yekutieli method and cut-off was set to q<0.01 in (**B**) and (**E**). Mann–Whitney *U*-test and false discovery rate analysis (classical 1-stage method) were used to compare data in (**D**); ****p<0.0001. UC remission, n=10; UC active, n=20.

**Abbreviations**: PC, principal component; UC, ulcerative colitis.

Next, we compared gene expression of the colonic mucosa between UC patients with active disease and UC patients during remission. A PCA based on the transcriptional profiles from the groups revealed two distinct clusters (Figure 4A). In total, 405 genes were upregulated and 41 genes were downregulated in UC active compared with UC remission (q<0.01; Figure 4B). Of note, a PCA including all UC patients and the healthy subjects clustered UC remission closer to the healthy cohort than to UC active (Supplementary Figure 3).

Analysis of the pathway scores in an OPLS-DA for UC active vs UC remission revealed a strong separation between the groups (*p*<0.0001; Figure 4C), with most pathway scores again increased in active disease, except for the autophagy pathway, which had a decreased score compared with remission (Figure 4D). Further analysis of the genes participating in the autophagy pathway revealed that *BECN1* and *MAP1IL3A*, related to autophagosome nucleation, were downregulated, whereas genes associated with activation of autophagosome complex (*ATG13, ULK1*) and sequestration processes (*ATG4K*) were upregulated in active UC (Figure 4E). In summary, active UC had a distinct gene expression signature compared with UC remission, including dysfunctional autophagy signaling.

### The Gene Expression of the Colonic Mucosa of UC Patients in Remission is Characterized by Dysfunctional Homeostasis

**Figure 5:**
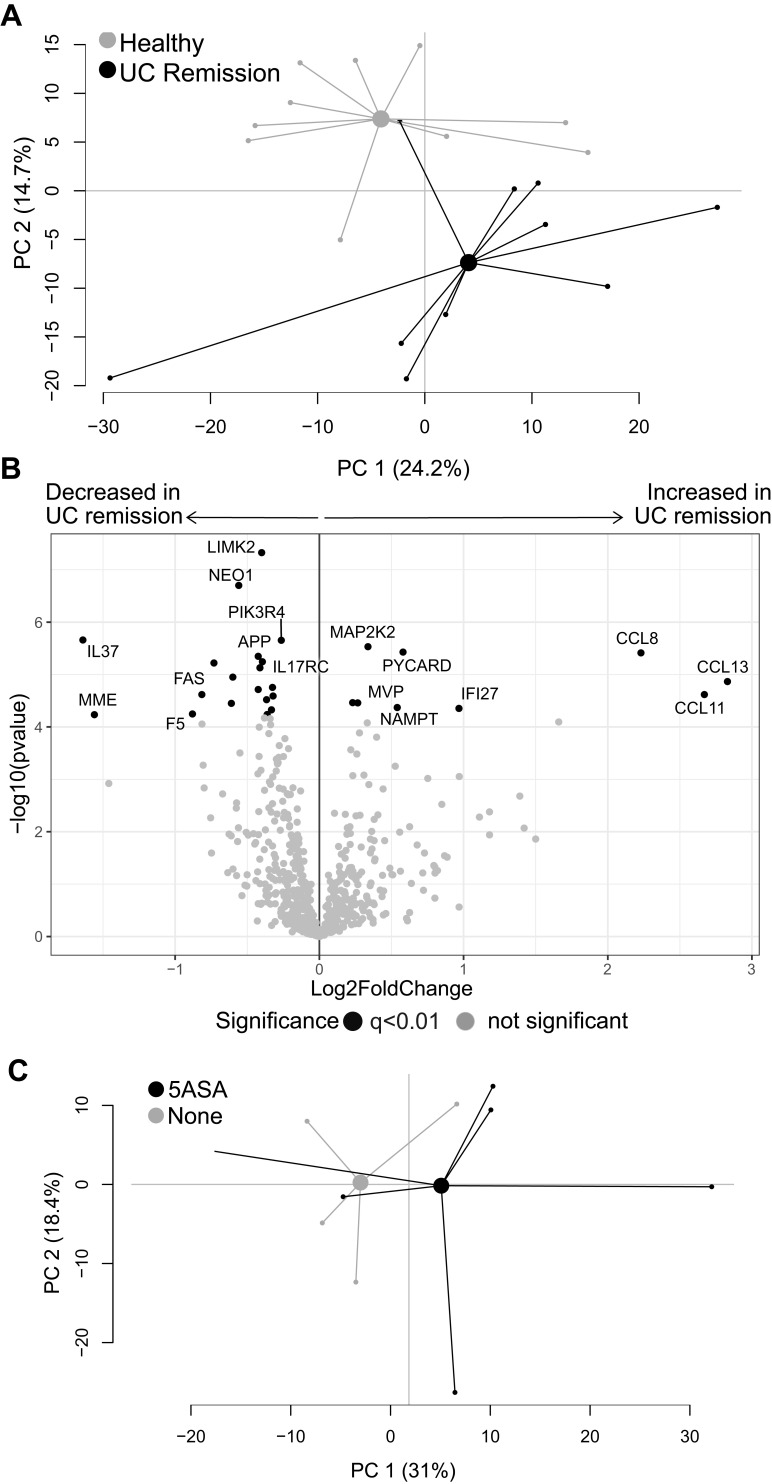
Gene expression analysis in UC patients in remission vs healthy subjects. Mucosal gene expression data in sigmoid biopsies from UC patients in remission and healthy subjects were analyzed using the NanoString nCounter Host response panel, 776 genes. (**A**) Principal component analysis for UC remission and healthy. (**B**) Volcano plot showing differential gene expression between UC remission vs healthy displayed as log_2_ fold change vs significance (Student’s *t*-test). False discovery rate analysis was performed using Benjamini–Yekutieli method and cut-off was set to q<0.01. (**C**) Principal component analysis for UC patients in remission grouped according to ongoing treatment; 5-ASA and no treatment. The volcano UC, ulcerative colitis; UC remission, n=10; healthy, n=10; 5-ASA, 5-aminosalicylic acid, n=6; No treatment, n=4.

**Abbreviations**: PC, principal component; UC, ulcerative colitis.

Lastly, we compared colonic gene expression between UC patients in remission (all with Mayo Score = 0) and healthy subjects. A PCA analysis of all genes revealed distinct separation between the groups (Figure 5A), and differential gene expression analysis showed 9 upregulated and 19 downregulated genes in UC remission compared with healthy subjects (*q*<0.01; Figure 5B).

A separate PCA was performed to investigate possible gene expression differences driven by treatment among patients with UC in remission, but no clear separation was observed between patients with or without ongoing 5-ASA treatment (Figure 5C).

**Figure 6:**
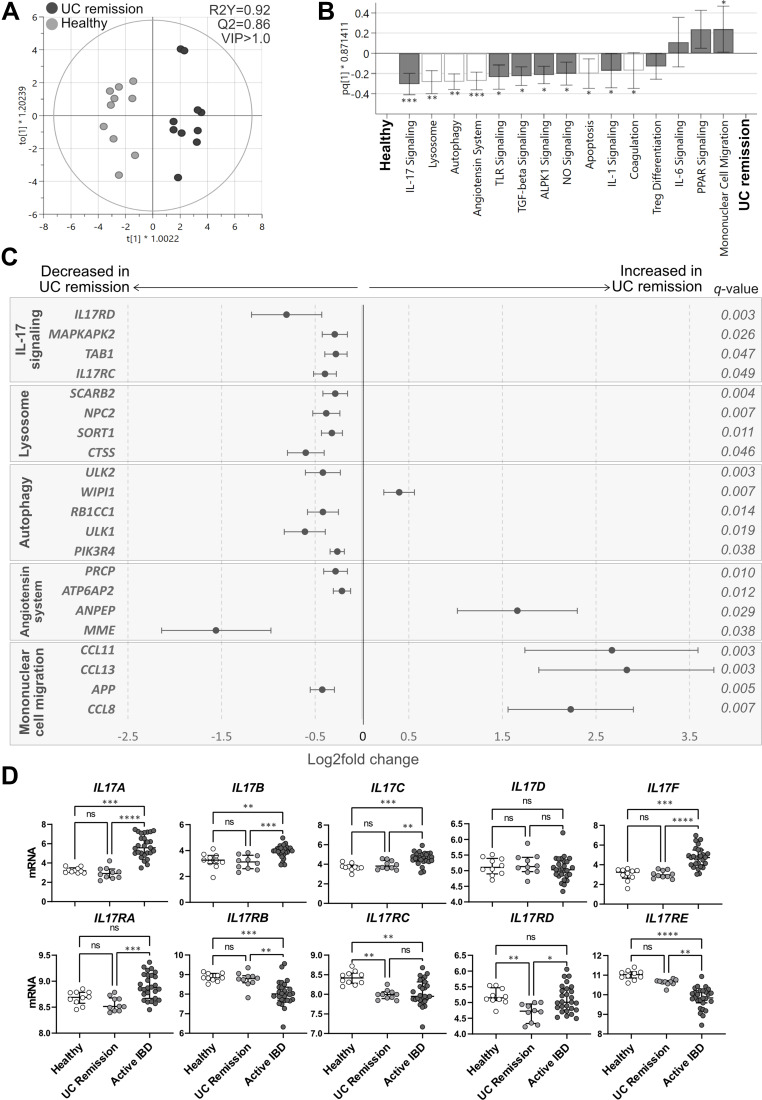
Pathway analysis in UC remission vs healthy subjects and mucosal expression of IL-17 cytokines and related receptors. Total RNA from mucosal biopsies was collected from sigmoid colon from UC patients in remission and healthy subjects. Expression of 776 genes was measured using the NanoString nCounter Host response panel and pathway scores for 56 pathways were generated. (**A**) Orthogonal partial least squares discriminant analysis (OPLS-DA) score scatter plot based on pathway scores after VIP>1.0 selection. (**B**) OPLS-DA loading column plot of pathway scores showing the included pathways after VIP>1.0 selection. Pathways shown with white bars belong to the phase of homeostasis (see Supplementary Table 1). Mann–Whitney *U*-test and false discovery rate analysis (classical 1-stage method) were used to compare the data; *q<0.05, **q<0.01, ***q<0.001. (**C**) Differentially expressed genes participating in the pathways IL-17 signaling, lysosome, autophagy, angiotensin system and mononuclear cell migration are shown in a forest plot based on log_2_ fold change. Error bars represent 95% confidence intervals. Student’s *t*-test was used to calculate significance between the groups (p-values) and false discovery rate analysis was performed using the Benjamini–Yekutieli method (q-values), cut-off was set to q<0.01. (**D**) Mucosal gene expression of IL-17 cytokines (top panel) and receptors (lower panel) from IBD patients with active disease, UC patients in remission and healthy individuals. Statistical difference between the groups was assessed by the Kruskal–Wallis test followed by Dunn’s multiple comparisons test. *p<0.05; **p<0.01; ***p<0.001; ****p<0.0001. UC, ulcerative colitis; UC remission, n=10; healthy, n=10; Active IBD, active inflammatory bowel disease, n=27.

The analysis of the pathway scores showed a separation between UC remission and healthy subjects in an OPLS-DA (*p*<0.0001; Figure 6A) and identified 11 pathways with decreased scores and one pathway with increased score in UC remission (Figure 6B). Interestingly, five out of the 11 pathways with decreased scores were linked to homeostasis (indicated with white bars in Figure 6B). In depth analyses of the genes participating in the most affected pathways revealed an upregulation of chemokines (*CCL11, CCL13, CCL8*), an angiotensin system-related gene (*ANPEP*) and an early regulator of autophagosome assembly (*WIPI1*) (Figure 6C). Remaining genes participating in angiotensin system (*PCRP, ATP6AP2, MME*), lysosome (*SCARB2, NPC2, SORT1, CTSS*), IL-17 receptor expression and signaling (*IL17RD, MAPKAPK2*, Table 1, *IL17RC*), autophagy (*ULK2, RB1CC1, ULK1, PIK3R4*) and mononuclear cell migration (*APP*) pathways were downregulated in UC remission compared with healthy subjects (Figure 6C). The gene encoding TGFb induced kinase, Table 1, participating in IL-17 signaling, also involved in the ALPK1 pathway was downregulated in UC remission compared to healthy subjects (q=0.027).

Individual analysis of gene expression of IL-17 related cytokines and receptors revealed an increase in the expression of *IL17A/B/C/F* and a decrease in the expression of the receptors *IL17RB/E* in active IBD compared with healthy subjects and UC remission (Figure 6D). Mucosal expression of *IL17RA* and *IL17RD* was increased in active IBD when compared with UC remission. Further, gene expression of *IL17RD* was decreased in UC remission compared to IBD active and healthy subjects and *IL17RC* was consistently decreased in IBD, both active and UC remission, when compared to the healthy subjects (Figure 6D, bottom panel).

In conclusion, despite a Mayo score of zero, the colonic mucosal gene expression in UC patients in remission differ from healthy subjects with emphasis on homeostasis.

## Discussion

This study demonstrates that active and quiescent disease share mucosal dysfunctionality related to autophagy, ALPK1 and IL-17 signaling pathways, although the majority of colonic mucosal gene expression and signaling pathway scores differed between the two phases. These results support the presence of permanent mucosal alterations, even when patients are in remission, and indicate that dysregulation of these pathways may potentially trigger future disease flares. However, we recorded no major differences in the mucosal gene expression between active colonic CD and UC.

Numerous genes and signaling pathways related to pro-inflammatory processes, microbiota sensing and stress mechanisms were upregulated in active IBD rather evenly, independent of biopsy site. As inflammation is well known to be linked to these processes, we instead focused on the pathways that rendered lower scores in active IBD, reflecting downregulated signaling cascades. Interestingly, lower pathway scores of autophagy, ALPK1, IL-17 and leukotriene/prostaglandin signaling were found in IBD patients with active disease compared to healthy subjects. Similarly, autophagy, ALPK1 and IL-17 signaling pathway scores were decreased in patients with quiescent UC compared to healthy subjects, suggesting that these pathways are consistently impaired in UC, and not related to disease activity per se.

Autophagy is important for removing waste cell components through a lysosome-dependent mechanism, but also has a major role in host defense against intracellular bacterial pathogens.^24^ Within the intestinal epithelium, impaired autophagy may allow for breaches of the epithelial barrier by both pathogenic and commensal bacteria due to defective antimicrobial granule formation and reduced mucus secretion.^25^ In our study, reduced autophagy pathway scores in IBD patients with active and quiescent disease was accompanied by downregulation of several pathway genes (*ULK2, RB1CC1, PIK3R4*) in both groups. Still, the mucosal expression of *NOD2*, known to direct autophagy by recruiting ATG16L1 to the plasma membrane at the site of bacterial entry,^26^ was increased during active disease but found at normal levels during remission. In quiescent UC, the lysosome pathway score was also decreased, suggesting impaired function of membrane-bound lysosomes crucial for breaking down waste products of autophagy. One of several downregulated genes was *CTSS*, which encodes cathepsin S, a lysosomal enzyme expressed by immune and epithelial cells in response to inflammatory mediators, regulating antigen presentation and taking part in degradation of extracellular matrix. Taken together, these results suggest that a NOD2–independent dysregulated autophagy is a perpetual feature of IBD unrelated to inflammation, and stress the importance of the proper function of this mechanism for intestinal homeostasis.

While the mucosal expression of the pro-inflammatory *IL17A/B/C/F* cytokines was increased in active IBD, it is indeed interesting to note that the pathway scores for the IL-17 signaling pathway, including expression of *IL17RC*, were consistently lower in active disease and remission as compared to healthy subjects. Furthermore, disruption in autophagic activity have been reported to favour TH17 differentiation and secretion IL-17 cytokines by enabling the release of IL-23 and IL-1 cytokines.^27^,^28^ However, no evidence for a dysregulated TH17 differentiation pathway was recorded in the present study. Signal transduction for IL-17A and IL-17F, mainly produced by activated TH17 cells, requires the presence of a heterodimeric complex consisting of the ubiquitously expressed IL-17RA and the inducible IL-17RC, and the absence of either receptor results in ineffective signaling.^29^ Thus, our data implicates an impaired mucosal IL-17A/F signaling in IBD, known to be important for host response and clearance of extracellular pathogens,^30^ irrespective of disease activity. The evidence of monoclonal antibody therapy targeting IL-17A triggering onset or exacerbation of IBD, further supports the notion that the IL-17 signaling pathway is crucial for maintaining gut homeostasis and prevents hyperactive innate inflammatory processes.^31^

The ALKP1 pathway was downregulated during active disease as well as in remission. ALKP1 is a cytosolic innate immune receptor for metabolic intermediates of LPS biosynthesis in Gram-negative bacteria and essential for activation of the NFkB protein complex.^32^ In animal models, ALKP1 promotes intestinal homeostasis by regulating the balance of TH1/17 immunity following microbial challenge and ALKP1 deficiency results in loss of anti-inflammatory properties and promotes colitis.^33^ Thus, the reduced ALKP1 signaling pathway score in our study suggests an impaired ability to maintain anti-inflammatory mechanisms, and thereby promote inflammation.

Also the leukotriene/prostaglandin pathway was deemed as downregulated in active IBD in our data set, suggesting reduced prostaglandin and leukotriene signaling. However, in depth characterization of the pathways’ genes that contributed to this somewhat unexpected finding, showed that *HPGD* and *EPHX2*, both enzymes crucial for breaking down the inflammatory mediators prostaglandin and epoxyeicosatrienoic acids, were reduced,^34^,^35^ while the enzymes *ALOX5, ALOX15* and *ALOX5AP* mediating the transition from arachidonic acid to leukotrienes were increased.^36^ Hence, this supports a role for increased activity of leukotriene and prostaglandin mediators in maintaining inflammation in patients with active disease.

Although UC patients in remission had an endoscopic Mayo score of zero, the mononuclear cell migration pathway score was increased, primarily driven by increased expression of *CCL8, CCL11* and *CCL13*, as compared to healthy subjects. The chemokine CCL11 (eotaxin-1) selectively recruits eosinophils, whereas CCL8 and CCL13 are chemotactic for and activate mast cells and eosinophils as well as monocytes and T cells. Our data suggest that during what is considered as clinically and endoscopically inactive disease, there is an active recruitment of inflammatory cells, with emphasis on eosinophils. Increased numbers of activated mucosal eosinophils have previously been reported in active and quiescent IBD,^37–39^ and an increased influx of eosinophils to the intestinal mucosa during quiescent disease may potentially be involved in subclinical inflammation and increased intestinal permeability as well as tissue healing.^40^

The Renin-Angiotensin System (RAS) is important for controlling the vascular tone as well as salt and fluid balance. In a cascade of events, angiotensinogen is cleaved to angiotensin (AT) II, activating numerous cell types including intestinal epithelial cells, immune cells and stromal fibroblasts.^41^ Although RAS has a major role in inflammation, fibrosis, immune cell recruitment and apoptosis, little is known about RAS in IBD.^42^ Still, overexpression of renin increases susceptibility to experimental colitis.^43^ In our study, the angiotensin system pathway score was downregulated in UC remission but not affected in active UC or CD. The mucosal expression of *ANPEP*, encoding for alanyl aminopeptidase converting the vasoconstrictor AT II to the less biologically active AT IV, was increased during remission. This was accompanied by decreased expression of *MME* encoding for Neprelysin and *PRCP* encoding angiotensin C, converting and breaking down AT II, and decreased expression of *ATP6AP2* encoding for the renin receptor.^44^ Thus, the pattern suggests downregulation of RAS, promoting less vasoconstriction in the mucosal tissue of UC patients in remission as compared to healthy subjects.

In our cohort, the mucosal gene expression overlapped between colonic CD and UC during active inflammation, ruling out the possibility to identify differentially regulated signaling pathways between the patient groups. Only *MGAM*, encoding the intestinal brush border Maltase-glucoamylase involved in starch digestion, tended to be differently expressed in UC and colonic CD. The result may reflect the lack of major differences in mucosal gene expression between UC and colonic CD, or the low number of CD patients included in the study.

We acknowledge that this study has limitations. The low number of study participants in the colonic CD study group, the restricted information on comorbidities, and the lack of a validation cohort, are limiting factors. We were only able to analyze CD patients in the context of active disease since no CD patients in remission were included, which restricted the possibility to compare IBD in remission as a whole. Furthermore, UC patients in remission were independently included from the active UC group, which did not allow paired analyses and evaluation of within-subject effects, and the number of individuals in each treatment group limits the generalizability of our results. We also recognize that sampling site bias may have affected our results to some extent, nevertheless no substantial intestinal regional variations were observed for the panel used in this study. Therefore, limiting this study to only colon samples may be considered a strength, allowing characterization of colonic disease during flares. Still, future studies including inflamed ileal samples are needed to understand site-specific transcriptional characteristics. Further, no differences in gene expression concerning pharmacological treatments were found, and none of the IBD patients were receiving biologic treatment at inclusion. Moreover, although the use of a targeted sequencing method avoids overinterpretation of the data, the chosen assay is limited to host immune response, and unrelated genes and pathways contemplated in other panels or in untargeted mRNA sequencing analyses are not represented here. Despite these limitations, the method used does not only determine gene expression of host susceptibility, interferon response, innate and adaptive immune responses and homeostasis, but also provides insights across more than 50 pathways rendering new knowledge of possible dysfunctional mechanisms of IBD, persistent during flares and remission.

## Conclusion

In summary, this study demonstrates that genes and pathways linked to autophagy, ALPK1 and IL-17 signaling are persistently downregulated in UC irrespective of disease activity. Further, UC patients in remission present a unique mucosal environment, suggesting dysfunctional mechanisms preventing patients from reaching and sustaining true homeostasis. These findings may enable better comprehension of the remitting and relapsing pattern of the disease and guide future studies to personalize therapeutic strategies to treat and prevent flares. Lastly, colonic CD and UC had an overlapping genetic signature, suggesting that colonic IBD phenotypes share major immune characteristics.

## Data Sharing Statement

The dataset from this publication is available from the corresponding author upon reasonable request.
